# Transcriptional evidence of reduced BDNF trophic capacity in the post-mortem human midbrain of schizophrenia cases with high inflammation

**DOI:** 10.1038/s41398-025-03359-7

**Published:** 2025-05-07

**Authors:** Jessica J. Chandra, Yunting Zhu, Alice Petty, Yasmine Kostoglou, William X. Haynes, Maree J. Webster, Cynthia S. Weickert

**Affiliations:** 1 Schizophrenia Research Laboratory, Neuroscience Research Australia, Randwick, NSW 2031 Australia; 2 School of Psychiatry, Faculty of Medicine, University of New South Wales, Sydney, NSW 2052 Australia; 3 Department of Neuroscience & Physiology, Upstate Medical University, Syracuse, NY 13210 USA; 4 Laboratory of Brain Research, Stanley Medical Research Institute, Rockville, MD 20850 USA

**Keywords:** Molecular neuroscience, Schizophrenia

## Abstract

Elevated inflammation in the midbrain of ~45% of people with schizophrenia may relate to altered trophic support for neurons. Dopamine neurons require trophic support from Brain-Derived Neurotrophic Factor (BDNF), that signals via the full-length Tropomyosin kinase B receptor (TrkB^TK+^). The truncated BDNF receptor (TrkB^TK-^) and the apoptosis-related p75 receptor may counteract the effects of BDNF. We hypothesised that transcriptional changes in either BDNF, and/or a transcription factor critical for the maintenance of dopamine neurons (Nuclear Receptor Related-1 protein; NURR1), and/or BDNF receptors – TrkB (TK+ or TK-) and p75, would be found in the post-mortem schizophrenia midbrain, particularly in schizophrenia cases defined as “high inflammation”. The neuroinflammatory status was delineated based on elevated expression levels of a combination of pro-inflammatory transcripts (SERPINA3, IL6, IL1β and TNFα) and defined as a subgroup (46%) by 2-step recursive clustering. Using RT-qPCR, mRNA levels of NURR1, BDNF, TrkB and p75 was quantified in schizophrenia (n = 65) and control (n = 64) ventral mesencephalon. We found significant decreases in BDNF, TrkB^TK+^ and NURR1 (14–18%) and increases in TrkB^TK-^ and p75 (18–35%) mRNA levels in schizophrenia compared to controls (all *p* < 0.05), with exacerbation of changes identified in high inflammation schizophrenia. To determine whether these changes would be consistent with resulting from chronic antipsychotic treatment, we treated healthy adult rats with antipsychotics (haloperidol and risperidone) for 7 months and found all transcripts to be unaltered compared to control rats. SnRNAseq of human midbrain showed that p75 receptor mRNA is primarily localised in oligodendrocytes and pan-TrkB mRNA is in both neurons and astrocytes. We confirmed that p75 was localised to oligodendrocyte-like cells by immunohistochemistry. Altogether, we find transcriptional evidence of reduced trophic support in schizophrenia midbrain and suggest that this may directly impact dopamine neuron health, particularly when neuroinflammation is also present.

## Introduction

Psychotic symptoms of schizophrenia are believed to arise from excessive dopamine production in the mesolimbic pathway projecting from the ventral tegmental area to the ventral striatum [1, 2], which is supported by the specificity of antipsychotic action in limbic regions [3, 4]. Recent studies have demonstrated dopamine dysfunction further extends to other regions of the midbrain, encompassing excessive dopamine production by nigrostriatal dopamine neurons projecting from the substantia nigra to the dorsal striatum [5, 6]. The higher energetic demands of increased dopamine release in schizophrenia may reduce the health, plasticity and viability of midbrain dopamine neurons. The excess dopamine may contribute to an increase in reactive oxygen species, which are known to disrupt cellular homeostasis [7] and induce neuroinflammation. Indeed, we used two-step recursive cluster analysis to define a high inflammatory biotype of ~46% of schizophrenia cases based on elevated pro-inflammatory transcripts (SERPINA3, IL6, IL1β, TNFα) in the midbrain across two independent cohorts [8, 9]. This increase in brain tissue inflammation would also be consistent with compromised dopamine neuron health leading to a need for more trophic support. Dopamine neurons rely upon trophic factors such as Brain-Derived Neurotrophic Factor (BDNF) for proper development, for maturation and for survival [10–13]. BDNF is synthesised as preproBDNF, which is cleaved into proBDNF and mature BDNF [14]. Mature BDNF binds to the full-length Tropomyosin Kinase B receptor (TrkB^TK+^) localised in neurons [15, 16] and to the truncated Tropomyosin Kinase B receptor (TrkB^TK-^) localised mainly in glia [17, 18]. TrkB^TK+^ activates pro-survival signalling cascades through phosphorylation of the intracellular tyrosine kinase domain [19, 20]. Co-expression of p75 with TrkB^TK+^ in neurons promotes BDNF binding affinity [21]. While the p75 receptor can also bind to proBDNF to elicit apoptotic signalling [22, 23], the cellular localization of p75 in dopamine neurons in the human midbrain has not been established, so distinguishing between a faciliatory role or a deleterious role for p75 in terms of midbrain BDNF is challenging.

Significant reductions in BDNF and TrkB^TK+^ (mRNA and protein) are present in multiple brain regions in schizophrenia, including the dorsolateral prefrontal cortex (DLPFC), anterior cingulate cortex and hippocampus [15, 24–30]. We hypothesised that these reductions in BDNF and TrkB^TK+^ mRNA also exist in the ventral midbrain in schizophrenia, a region of concentrated dopamine neurons [31, 32]. BDNF expression is regulated by the transcription factor Nuclear Receptor Related 1 (NURR1), which induces the dopaminergic phenotype and maintains survival cues for dopamine neurons [33, 34]. We sought to determine whether NURR1 mRNA is reduced in schizophrenia, potentially providing further evidence that there may be a reduction in health along with less trophic signalling for dopamine neurons in the midbrain.

The interaction between neuroinflammation and trophic signalling in the midbrain in schizophrenia may also involve changes in the truncated isoform of the TrkB receptor (TrkB^TK-^). This form of TrkB lacks the tyrosine kinase domain, and is predominantly localised in resident immune cells of the brain – astrocytes - and to a lesser extent, microglia [16, 18, 35]. TrkB^TK-^ expression on immune cells promotes gliosis and cytokine release, and diverts BDNF-mediated trophic support away from neurons [17, 36]. Additionally, TrkB^TK-^ can form dimers with TrkB^TK+^ in neurons, where TrkB^TK-^ functions as a dominant-negative inhibitor to block pro-survival signalling [17, 37, 38]. TrkB^TK-^ mRNA is increased in the DLPFC in schizophrenia [39], and a similar percentage of people with schizophrenia display elevated pro-inflammatory markers (~40%) in the cortex [40]. However, it is unclear whether these increases in TrkB^TK-^ mRNA are concentrated in the subset of high inflammatory schizophrenia cases, or whether they occur more generally.

Hyperdopaminergia is evident in the midbrain in schizophrenia, and since inflammation may interact with BDNF to alter neuron plasticity and function, we hypothesised that BDNF and TrkB^TK+^ mRNA would be reduced, whilst TrkB^TK-^ mRNA would be increased in the midbrain in schizophrenia, particularly in high inflammation conditions. We further hypothesised that the apoptotic p75 receptor and NURR1 transcription factor would be changed in the schizophrenia midbrain and these changes may relate to neuroinflammation. In order to determine the cellular source of BDNF, TrkB and p75, we interrogated our single-nucleus RNA sequencing (snRNAseq) results from the post-mortem human midbrain [41]. Additionally, the localisation of the p75 receptor protein was established using immunohistochemistry to determine whether this receptor could mediate BDNF actions on midbrain dopamine neurons or another cell type. To test whether changes in neurotrophins may result from chronic antipsychotic treatment, we chronically administered typical and atypical antipsychotics to healthy adult male rats and then assessed gene expression of trophic signalling molecules in the substantia nigra.

## Methods

### Post-mortem human cohorts/midbrain tissue collection and defining inflammatory subgroups

All experimental protocols using human brain tissue were approved by the University of New South Wales (UNSW) Human Research Ethics Committee (HREC#12435). Diagnosis for schizophrenia was made according to DSM-IV and cases were matched with controls individually on the basis of age, sex, RNA Integrity Number (RIN), and post-mortem interval (PMI) between schizophrenia cases and controls, which did not differ overall in either cohort alone or in the combined cohort, whereas brain pH was significantly lower in schizophrenia cases compared to controls (Table 1). All but 2 schizophrenia patients were known to have received antipsychotic treatment. Where available, antipsychotic measurements (mean daily, lifetime and last dose) were converted to chlorpromazine (CPZ) equivalents and are recorded with duration of illness in schizophrenia patients (Table 1, Table S1–3). In the combined SMRI and TRC cohort, 45 schizophrenia patients were treated with typical antipsychotics, 18 were treated with atypical antipsychotics, and the clinical medication history of 2 cases were unknown.

**Table 1:** Demographic profiles of schizophrenia and control groups from a combined NSW Brain Tissue Resource Centre and Stanley Medical Research Institute cohort used for gene expression analysis.

qPCR cohort	Control group (n = 61)	Schizophrenia group (n = 63)	Statistics
Age in years (range)	47.3 ± 9.9 (22–67)	46.5 ± 10.8 (24–67)	*t*(124) = 0.501, *p* = 0.617
Sex (Male/Female)	45/16	45/18	–
pH	6.6 ± 0.3	6.5 ± 0.2	***t***(**124**) = **3.356,** ***p*** = **0.001**
PMI (hours)	30.4 ± 11.8	33.3 ± 16.5	*t*(124) = −1.055, *p* = 0.293
RIN	5.8 ± 1.2	6.0 ± 1.3	*t*(124) = −0.837, *p* = 0.404
Manner of death (Natural/Suicide)	61/0	49/14	–
Duration of illness (years)	—	24.6 ± 11.9	–
Lifetime CPZ equivalent (g)	—	3229.2 ± 6670.2	–
Smoking around time of death	Yes = 23, No = 21, Unknown = 17	Yes = 41, No = 10, Unknown = 12	–

Group sizes vary as 3 cases were excluded due to low RNA Integrity Number (RIN < 3), 1 case was excluded due to failure of complementary DNA (cDNA) synthesis and another 2 control cases were removed due to immune-related illnesses. Data are presented as mean ± SD. Bold test indicates statistically significant data.

For homogenate-based work, post-mortem midbrain tissue was provided by the NSW Brain Tissue Resource Centre (TRC, Sydney, Australia) (n = 30 schizophrenia and n = 30 controls) and Stanley Medical Research Institute (SMRI, Maryland, USA) (n = 35 schizophrenia and n = 35 controls) (Table 1; Table S1). Sample size for post-mortem human brain cohorts was calculated to detect a mean change of 20% using the average standard deviation for each transcript. For adequate power (0.8), n = 130 samples were sufficient. Controls were screened to ensure no history of neurologic or psychiatric disorder based on information about development, lifestyle, family history of mental illness, drug and alcohol use, smoking and medical problems obtained through a structured telephone interview with a first-degree relative. Ventral midbrain tissue was dissected from six 60 μm cryostat sections cut in the coronal plane at the level of the oculomotor nerve exit.

For defining inflammatory subgroups, we relied on our previous studies employing RNA sequencing of post-mortem human dorsolateral prefrontal cortex tissue followed by pathway analysis that revealed that the GO category with the most increased transcripts in schizophrenia compared to controls were inflammatory pathways. Diagnostic changes were then confirmed by RT-qPCR [40]. Levels of three pro-inflammatory cytokines and one acute phase protein (IL1β, IL6, IL8, SERPINA3) were identified as the most distinct changes and were used to define 2 inflammatory groups: a normative or “low” inflammation subgroup and a “high” inflammation subgroup with two-step recursive clustering methods. All the mRNAs identified as relating to inflammation in the cortex were evaluated in the midbrain, and unbiased clustering identified a largely overlapping set of transcripts; SERPINA3, IL6, IL1β, and TNFα, as the cytokines most predictive of inflammatory subtype in the midbrain. These four mRNAs were used to define 2 inflammatory subgroups (“high” and “low”) across two post-mortem human midbrain cohorts [8, 9]. A subgroup of cases from the SMRI cohort was used for snRNAseq to determine the cellular distribution of BDNF, TrkB and p75 mRNA expression (14 controls, 20 schizophrenia cases) (Table S2). The TRC cohort was used for immunohistochemical analysis of p75 receptor protein expression in the midbrain (Table S3).

### Animal treatment and tissue extraction

All experimental protocols on animals were approved by the UNSW Animal Research Ethics Committee (#21/26 A). Adult male Sprague Dawley rats (Animal Resource Centre, WA, Australia) were habituated from P60 to twice daily treatment with drug-free cookie dough for 1 week. Each cage of animals was then randomly assigned treatment to either cookie dough with 2 mg/kg/day haloperidol, or 2 mg/kg/day risperidone added, or control cookie dough without antipsychotic (n = 14/group). Sample size for the rodent cohort was estimated to detect an effect size of 0.5 with power = 0.8 and alpha = 0.05 (G*Power 3.1). Antipsychotic doses were based on conversion factors applied to human doses stipulated in Australian Medicines Handbook guidelines (based on 60 kg average human weight) and maximum D2 receptor occupancy [42, 43]. Previous studies utilising equal or lower doses of haloperidol and risperidone also report behavioural and physiological effectiveness of these doses [44–48].

Rats were weighed weekly and cookies were made to respective weights ±10%. Haloperidol (Serenace 5 mg tablets, Aspen Pharma Australia) and Risperidone (Rixadone 4 mg tablets, Alphapharm) were crushed for supplementation in cookie dough [35% cornflour, 10% gelatine powder, 35% icing sugar, 20% milk powder dissolved in MilliQ water (17% mL/g)] [46, 49, 50] and given twice daily for 7 months to resemble the extended antipsychotic exposure that humans with schizophrenia would receive relative to their corresponding life-span (mean ~20 years duration of treatment in humans [51]). Following the treatment period, animals were anaesthetised with isoflurane in a gas chamber prior to decapitation. Brains were excised, sectioned coronally, snap frozen in isopentane (Sigma-Aldrich) and stored at −80 °C. To extract midbrain tissue, the midbrain block was faced on the cryostat until the anterior landmark for the substantia nigra was visualised around bregma −4.56 mm [52]. Ventral midbrain tissue was dissected from five 100μM thick sections for RNA extraction. Experimental procedures including behavioural assessment, brain dissections, RNA extraction and analyses of transcripts was conducted blinded with animals coded with randomly allocated numbers by an independent researcher.

### RNA extraction and quantitative real-time polymerase chain reaction (qPCR) analysis

Midbrain tissue from human (19–83 mg) and rodent samples (~20 mg) was homogenised in TRIzol (Life Technologies, Scoresby, VIC, Australia). Quantity and quality of total RNA was determined using Nanodrop ND 1000 spectrophotometer (Nanodrop Technologies, Wilmington, DE, USA). RIN was measured with an Agilent 2100 Bioanalyzer (Agilent Technologies, Santa Clara, CA, USA). Superscript IV First Strand Synthesis Kit (Life Technologies) and random hexamers were used to synthesise cDNA, following the manufacturers’ instructions. TaqMan gene expression assays (Applied Biosystems, Life Technologies, Foster City, CA, USA) were used for qPCR analysis of BDNF IV, NURR1, TrkB^TK+^, TrkB^TK-^ and p75 in the human (Table S4) and rodent cohort (Table S5). BDNF exon IV was assessed due to its relatively higher expression level in the substantia nigra compared to other exons and because of its activity-dependent transcription [53, 54]. Human gene expression was measured in a 1:5 dilution of cDNA using high-throughput integrated fluidic circuits by Fluidigm Biomark HD (Fluidigm Corporation, CA, USA) at the Ramaciotti Centre for Genomics (UNSW, Sydney, Australia). A dilution series of cDNA pooled from all samples was included to determine gene expression in samples using the relative standard curve method (n = 8 points), as previously described [55]. Controls without template and reverse transcriptase were included on each plate. Fluidigm Real-Time PCR Analysis software version 4.5.2 (Fluidigm Corporation, CA, USA) was used to extract and analyse qPCR data, with the quality threshold set at 0.65. Genes of interest were normalised to the geomean of 4 housekeepers: beta-glucuronidase (GUSB), glyceraldehyde 3-phosphate dehydrogenase (GAPDH), TATA-box binding protein (TBP) and ubiquitin C (UBC), which were unchanged between diagnostic groups in the human cohort (*p* > 0.05; Fig. S1). For the rodent cohort, each sample was assayed in triplicate using the ABI Prism 7900HT System (Applied Biosystems, Life Technologies). Serial dilutions of cDNA pooled from all samples were included as 8 standards and the relative standard curve method was used to quantify gene expression using Sequence Detector Software (V2.4, Applied Biosystems). Genes of interest were normalised to the geomean of GAPDH, GUSB, β-actin (ACTB) and tyrosine-3-monooxygenase (YWHAZ) and were unchanged by treatment (*p* > 0.05; Fig. S2).

### SnRNAseq

Ventral midbrain tissue was extracted from five 100 μm sections of a sub-cohort of cases from SMRI. Tissue was lysed in a sucrose lysis buffer, filtered, and nuclei were purified by ultracentrifugation in a dense sucrose buffer, as previously described [41, 56]. Tissue quality was assessed from adjacent sections to sampled tissue using RNeasy Lipid Tissue mini kit (Qiagen, 75804). Fluorescence activated sorting of nuclei identified DAPI-positive nuclei, which were negative for the early transcription marker for oligodendrocyte differentiation Olig2 (Anti-OLIG2 clone 211F1.1 AF488 mouse mAB, Merck Millipore, MABN50A4) and were also NeuN depleted, with only ~1 out 6 DAPI-positive nuclei also positive for the neuronal marker NeuN (1B7 AF647 mouse mAB, NBP1-92693AF647, Novus Biologicals). Single nucleus cDNA libraries were constructed with Chromium Single Cell 3’ Reagents Kit v3 (10x Genomics), according to the user guide. Equimolar ratios of pooled samples were sequenced on a NextSeq 500 platform (Research Sequencing Facility, University Medical Centre, Groningen, Netherlands). Sequencing reads were processed and aligned to the GRCh38 human genome (release 110 downloaded from Ensembl ftp) using CellRanger 7.0.1, with both exonic and intronic reads included, along with basic quality control analyses using fastqc program [57]. The mean estimated number of nuclei was 5184 per sample and the mean total detected genes was 38,326. Count matrices filtered in CellRanger were input to R v4.3.2 with Seurat v5.0 [58]. Nuclei with >5% mitochondrial content were removed. Count information was log normalized and integrated with reciprocal principal component analysis using Seurat. Scrublet v0.2.1 was used to remove doublets with default settings. Unbiased clustering analysis was performed using Find Neighbors (dim=1:30, k.param=10) FindClusters function with resolution set to 0.4 and major cell types were manually defined based on the expression of marker genes (Table S6). Expression levels of specific genes examined in this study were graphed using feature plots in Seurat.

### Immunohistochemistry

Fresh frozen midbrain sections from all TRC cases were used to visualise p75 receptor protein expression. Slices (14μM) were cut using a cryostat (Leica 3050 M) at the level of the oculomotor nerve, collected onto gelatine-subbed slides, stored at −80 ^o^C and then thawed at RT for 20 min. Tissue was fixed in 4% paraformaldehyde in PBS, rinsed in PBS, then 30% MeOH with 3% H_2_O_2_ for 20 min to block endogenous peroxidase activity. Following additional PBS washes, slides were blocked with 10% normal horse serum (S2000, Vector Laboratories, CA, USA) diluted in PBS, 0.05% bovine serum albumin and 10% Triton for 1 hr. Primary antibody for p75 (1:800, rabbit anti-p75 ab52987, Abcam, VIC, AUS) was applied overnight at 4 ^o^C. Slides were rinsed in PBS and incubated with a 1:500 dilution of horse anti-rabbit IgG-biotinylated secondary antibody (BA200, Vector Laboratories) for 1 hr at RT. After rinsing and incubating with the avidin-biotin-peroxidase complex (PK4000, Vector Laboratories) for 1 hr at RT, 3,3’-diaminobenzidine solution (DAB D5637, Sigma-Aldrich, MO, USA; final concentration 12 mmol/L in PBS with 0.003% H_2_O_2_;) was applied for 2 min. A final PBS wash was followed by dehydration with an ethanol gradient and Nissl counterstaining (0.02% thionine), prior to coverslipping. Slides were visualised under a Nikon Eclipse 80i light microscope using the 20X objective and brightfield settings.

### Statistical analysis

Statistical tests were performed using SPSS (V26, IBM, Armonk, NY, USA), with the threshold for statistical significance set to *p* < 0.05. All experiments on each sample were conducted once and adjustments were not made for multiple comparisons. Outliers greater than two standard deviations from the diagnostic and immune subgroup mean in the human cohort and from treatment group mean in the rodent cohort were excluded separately for each transcript. Shapiro-Wilk and Levene’s test assessed for normality and homogeneity of variance of mRNA levels respectively within diagnostic and immune subgroups in human cohorts and within treatment groups in the rodent cohort. If data did not satisfy normality in any group, data was log_10_ transformed and re-tested for normality. Pearson’s and Spearman’s correlations were used to test any association between demographic variables with mRNA levels in human cohorts. As altered pH is implicated in the schizophrenia disease state, pH was not used as a covariate [59]. Where clinical history was available, Spearman’s correlations between gene expression with lifetime, daily and last CPZ-equivalent antipsychotic dose as well as duration of illness were calculated for schizophrenia cases. ANCOVA and Quade’s rank analysis was used to analyse normally and non-normally distributed gene expression data respectively with covariates. Where there were no covariates, Independent Samples t-test was used to test for diagnostic differences and ANOVA to test for immune subgroup differences, with Fisher’s Least Significant Difference *post-hoc* test, to assess gene expression in the human cohort. Where normality was not achieved, non-parametric Mann-Whitney *U* was used for comparison of mRNA levels by diagnosis or Kruskal-Wallis tests were used to compare mRNA levels between immune subgroups of the human cohort or by treatment group in the rodent cohort. Graphs were generated using GraphPad Prism 9 (GraphPad Software, La Jolla, CA) and data was plotted as mean ± SEM.

## Results

### Reductions in BDNF IV, NURR1 and TrkB^TK+^ and increases in TrkB^TK-^ and p75 mRNAs are found in the midbrain in schizophrenia

**Fig. 1:**
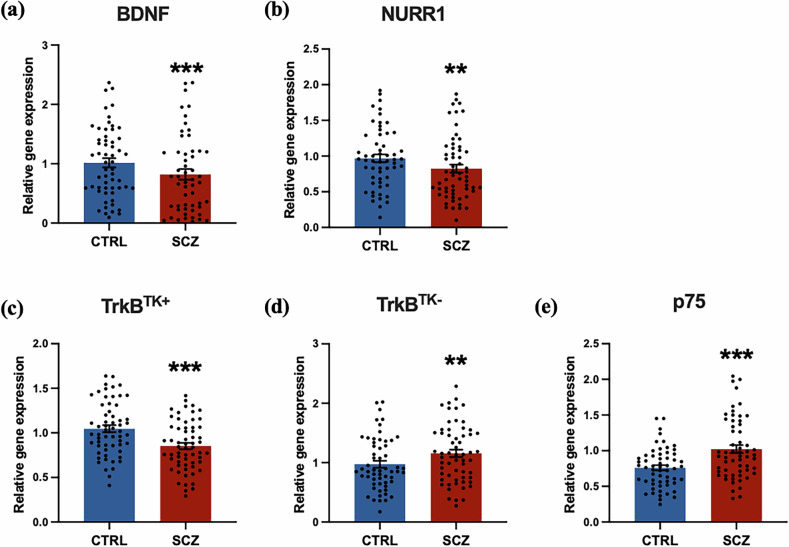
Gene expression of BDNF, NURR1, TrkB^TK+^, TrkB^TK-^ and p75 is altered in the human post-mortem schizophrenia midbrain. (**a**) Gene expression of BDNF and its (**b**) upstream transcription factor NURR1 is reduced in schizophrenia cases (n = 63; red) compared to controls (n = 61; blue). (**c**) Full-length TrkB^TK+^ receptor mRNA is also reduced, whereas mRNA of (**d**) the truncated TrkB^TK-^ receptor isoform and (**e**) apoptotic p75 receptor mRNA is increased in schizophrenia cases compared to controls. All data are presented as mean ± SEM, with individual data-points representing each case analysed (**p ≤ 0.01, ***p < 0.001).

Gene expression of BDNF IV and its upstream transcription factor, NURR1 was reduced by 18% [*F*_(1110)_ = 7.591, *p* = 0.007] and 14.9% [*F*(_1115_) = 6.867, *p* = 0.01] respectively in the schizophrenia group compared to controls (Fig. 1a, b). Gene expression of the primary full-length neurotrophic receptor of BDNF, TrkB^TK+^, was also reduced in schizophrenia cases compared to controls by 18.5% [*F*(_1116_) = 19.768, *p* < 0.001] (Fig. 1c). In contrast, mRNA levels of the truncated TrkB^TK-^ isoform were increased by 18.6% in schizophrenia cases compared to controls [*F*(_1117_) = 7.499, *p* = 0.007] (Fig. 1d). The p75 receptor mRNA levels were also increased by 34.7% [*F*(_1114_) = 19.89, *p* < 0.001] in schizophrenia cases compared to controls (Fig. 1e), reflecting the greatest magnitude of change evident across diagnostic groups of the receptors assessed.

### Diagnostic reductions in BDNF IV, NURR1 and TrkB^TK+^ and increases in TrkB^TK-^ and p75 mRNAs are magnified in high inflammation cases

**Fig. 2:**
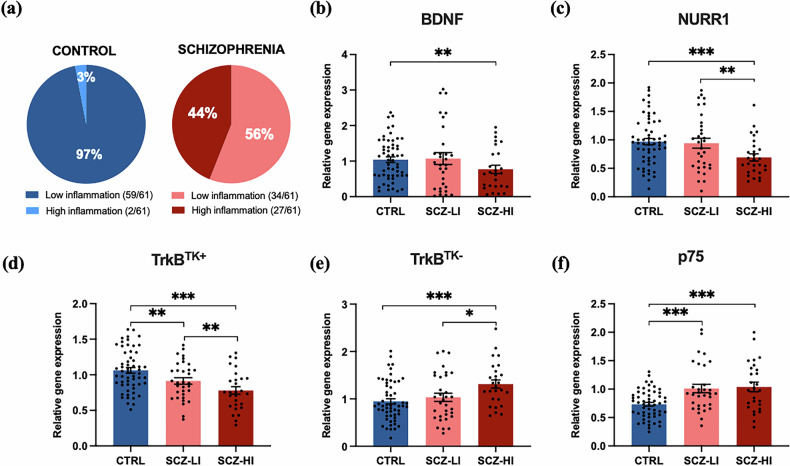
Marked changes in BDNF IV, NURR1, TrkB^TK+^, TrkB^TK-^ and p75 mRNA present in the post-mortem human midbrain of schizophrenia cases with elevated inflammation. (**a**) Two-step recursive cluster analysis of mRNA levels of immune markers (SERPINA3, IL6, IL1β and TNFα) yields immune subgroups: schizophrenia cases with low (pink) and high (red) immune markers, and controls with low (dark blue) immune marker mRNA expression. Significant reductions in (**b**) BDNF IV, (**c**) NURR1 and (**d**) TrkB^TK+^, as well as increased (**e**) TrkB^TK-^ and (**f**) p75 mRNA levels were evident in schizophrenia cases with a high inflammatory profile (SCZ-HI) compared to low inflammatory schizophrenia cases (SCZ-LI) and controls (CTRL). All data are presented as mean ± SEM, with individual data-points representing each case analysed (*p < 0.05, **p < 0.01, ***p < 0.001).

BDNF IV [*F*(_2110_) = 3.893, *p* = 0.023], NURR1 [*F*(_2111_) = 6.283, *p* = 0.003], TrkB^TK+^ [*F*(_2113_) = 15.07, *p* < 0.001], TrkB^TK-^ [*F*(_2113_) = 5.959, *p* = 0.003] and p75 [*F*(_2112_) = 11.624, *p* < 0.001] mRNA levels were also significantly changed according to inflammatory subgroups (Fig. 2). More specifically, there was a greater mean decrease in BDNF IV (30.8%; *p* = 0.007), NURR1 (30.1%; *p* = 0.001) and TrkB^TK+^ (23.5%; *p* < 0.001) mRNA levels when comparing high inflammation schizophrenia cases to low inflammation controls than when just examining diagnostic changes alone. TrkB^TK+^ mRNA was also decreased in low inflammation schizophrenia cases compared to controls (*p* = 0.011). However, the levels of BDNF and NURR1 mRNAs in the low inflammation schizophrenia group compared to low inflammation controls were not significantly different. A similar pattern of magnified changes in neuroinflammatory schizophrenia was observed for transcripts that were increased in the schizophrenia midbrain, where those with a high inflammatory status had a 46.3% increase in p75 levels (*p* < 0.001) and 38% increase in TrkB^TK-^ levels (*p* = 0.001) mRNA compared to controls. TrkB^TK-^ mRNA was also increased in the high inflammation schizophrenia subgroup compared to the low inflammation schizophrenia subgroup (*p* = 0.019). P75 mRNA levels were also significantly increased in the low inflammation schizophrenia subgroup compared to low inflammation controls (*p* = 0.001). Consistent with actions of the pro-trophic ligand and receptor pair, and the survival transcription factor, BDNF mRNA levels positively correlated with TrkB^TK+^ (*r* = 0.563) and NURR1 (*r* = 0.419) mRNA levels (both *p* < 0.0001) (Table S7). In contrast, BDNF mRNA levels negatively correlated with TrkB^TK-^ (*r* = −0.307) and p75 receptor (*r* = −0.362) mRNA levels (*p* < 0.001). P75 mRNA negatively correlated with NURR1 (*r* = −0.142, p = 0.014) and TrkB^TK+^ (*r* = −0.362, *p* < 0.0001) mRNA, yet p75 mRNA was not correlated with TrkB^TK-^ mRNA (*r* = −0.097, *p* = 0.293) (Table S7).

### Cell-type specific expression of BDNF, TrkB and p75 receptors

**Fig. 3:**
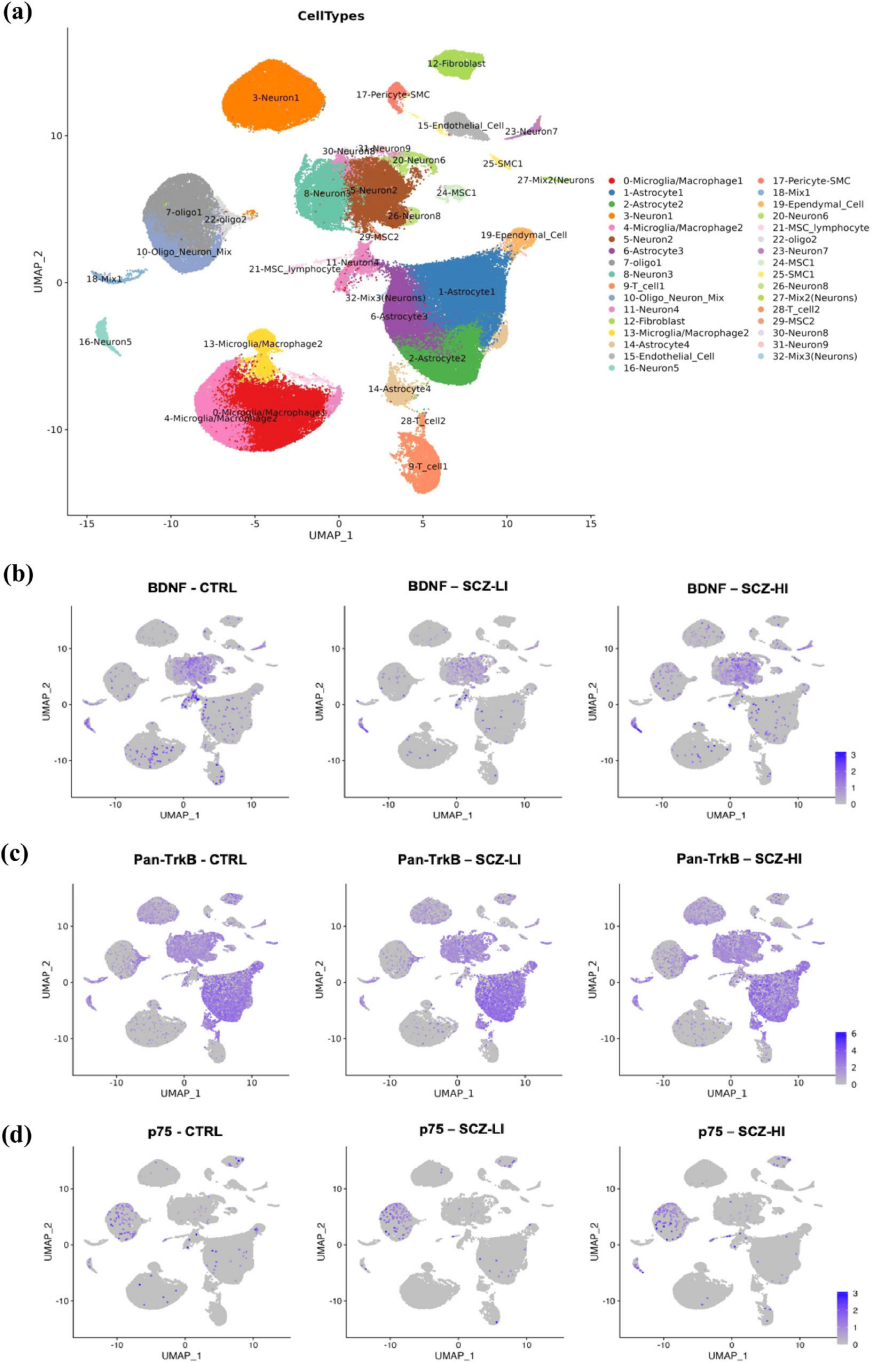
UMAP plots depicting expression of BDNF, p75 and TrkB mRNA across clusters of cells in the midbrain of schizophrenia cases with low and high inflammation and controls. (**a**) UMAP plot depicting manually defined cell types of clusters generated through unbiased clustering analysis of nuclei isolated from post-mortem human midbrain tissue from controls (n = 14) and schizophrenia (n = 20) cases. UMAP plots depicting gene expression across clusters of cells across immune subgroups shows (**b**) BDNF was predominantly localised within neurons in controls (CTRL-LI; n = 14) and schizophrenia cases with low (SCZ-LI; n = 10) and high (SCZ-HI; n = 10) inflammation. (**c**) Pan-TrkB receptor mRNA expression was predominantly evident in neurons and astrocytes, followed by endothelial cells and oligodendrocytes across both inflammatory subtypes of schizophrenia and controls. (**d**) P75 receptor mRNA was not evident in neurons and was primarily expressed in oligodendrocytes (black arrows) in controls, low and high inflammatory subtypes of schizophrenia cases.

SnRNAseq data of nuclei isolated from controls and schizophrenia midbrains were analysed together for unbiased cluster analysis of transcriptomic profiles from ~124,416 nuclei and yielded 33 clusters of cell types within the ventral midbrain as previously descripted (Fig. 3a) [41]. The top 10 transcripts used to characterise each cluster of cells are detailed in Table S6. Nuclei with similar transcriptomic profiles were grouped together, with some clusters containing multiple cell types. Neurons were the most prevalent cell type, spread across multiple clusters, followed by astrocytes, microglia/macrophages and oligodendrocytes (low proportion due to excluding Oligo2+ nuclei). Unbiased cluster analysis of the transcriptome of nuclei within control, low and high inflammation schizophrenia subgroups revealed a similar proportion of cells in each cluster. Comparison of BDNF gene expression within clusters across immune subgroups of schizophrenia cases and controls revealed that BDNF mRNA was primarily localised to neuronal clusters across all three subgroups, with occasional expression of BDNF evident in some astrocyte subtypes (Fig. 3b). High levels of BDNF mRNA were also observed in some microglia/macrophages in high inflammation schizophrenia cases, but not in low inflammation schizophrenia or controls. Pan-TrkB mRNA, comprised of both TrkB^TK+^ and TrkB^TK-^ isoforms, was robustly expressed across multiple cell types, with similar levels of expression in neurons and astrocytes across all three immune subgroups. Pan-TrkB expression was also evident in clusters containing fibroblasts, oligodendrocytes, endothelial cells, T cells and to a lesser extent microglia/macrophages and pericytes (Fig. 3c). In contrast, gene expression of the p75 receptor was of lower abundance and was limited to the cluster containing oligodendrocytes (Fig. 3d). Levels of p75 receptor expression was sparse and appeared to be marginally increased in several subtypes of oligodendrocytes in low and high inflammation schizophrenia subgroups compared to controls (Fig. 3d).

### P75 receptor protein is primarily localised to oligodendrocytes in the post-mortem midbrain

**Fig. 4:**
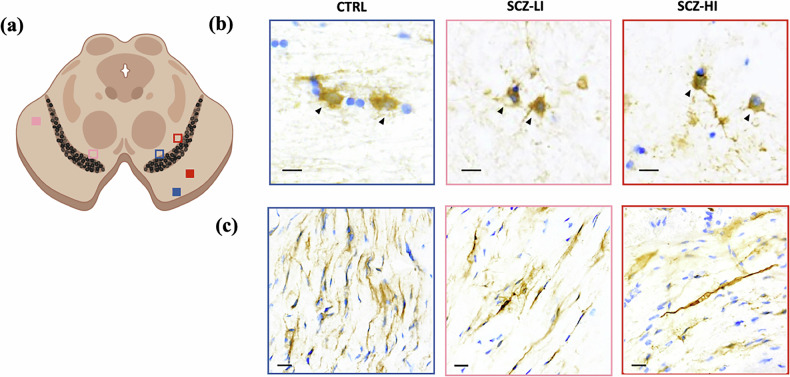
P75 receptor protein is expressed in oligodendrocytes in post-mortem human midbrain. (**a**) Representative image of the midbrain depicting where p75 receptor staining was visualised in relationship to the substantia nigra (dark region) in oligodendrocytes (square outlines) and in white matter tracts (filled squares) for control (CTRL, blue), low inflammation (SCZ-LI, pink) and high inflammation schizophrenia (SCZ-HI, red) cases. Image generated on Biorender. (**b**) Immunohistochemical staining of midbrain tissue obtained from CTRL, SCZ-LI and SCZ-HI cases reveals p75 receptor localisation within oligodendrocytes (black arrows). Scale bar = 10 μm. (**c**) P75 receptor protein was also evident in white matter tracts of the cerebral peduncles in a subgroup of schizophrenia cases, and to a lesser extent in controls. Scale bar = 20 μm.

Using immunohistochemistry, p75 protein was anatomically localised to the midbrain in all individuals in the TRC cohort (Fig. 4a). P75 receptor immunoreactivity was not evident in diffuse Nissl-stained nuclei of neuromelanin-containing dopamine neurons of the midbrain in either control or schizophrenia cases, nor was it found in neuron-like cells devoid of neuromelanin. However, in both schizophrenia and controls, the clearest signal for p75 protein in the midbrain was detected in putative oligodendrocytes, which were histologically identified as having a round cell body and lighter Nissl-stained nuclei compared to other glia-like cells, which are smaller and more intensely Nissl-stained [60, 61]. Oligodendrocytes positive for p75 immunostaining were most prominent in white matter of the midbrain sections (Fig. 4b). Dark p75 receptor immunoreactivity was evident in white matter tracts of the cerebral peduncles in 18% of controls (5/27 cases), 33% of low inflammation schizophrenia cases (5/15 cases) and 50% of high inflammation (6/12 cases) schizophrenia cases (Fig. 4c).

### Chronic antipsychotic treatment does not alter BDNF IV, NURR1, TrkB^TK+^, TrkB^TK-^ or p75 mRNA in the midbrain of healthy adult rats

**Fig. 5:**
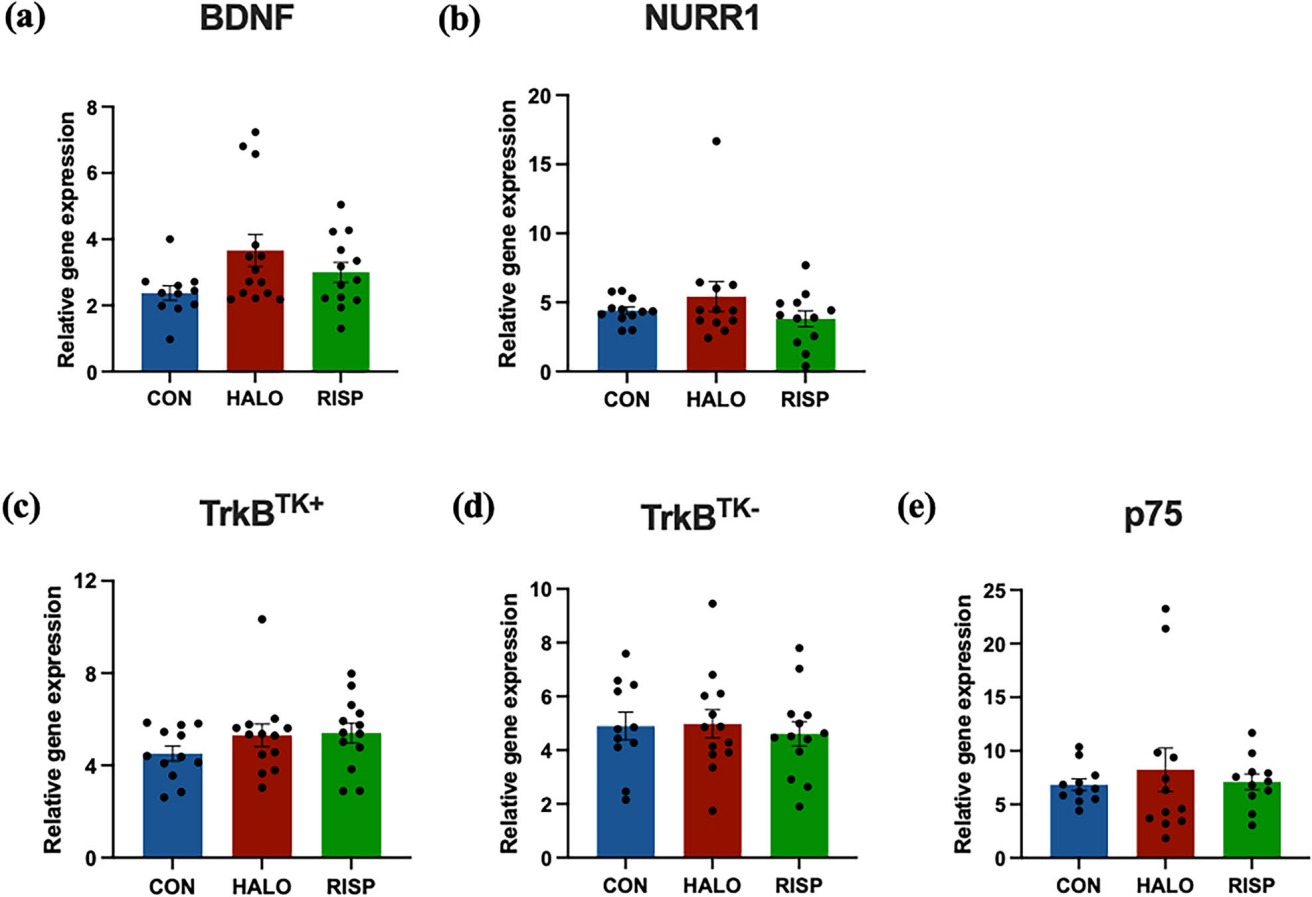
Chronic antipsychotic treatment does not alter BDNF trophic signalling capacity at the transcriptional level. Oral treatment with haloperidol (HALO) or risperidone (RISP) for 7 months does not change (**a**) BDNF IV, (**b**) NURR1, (**c**) TrkB^TK+^, (**d**) TrkB^TK-^ or (**e**) p75 mRNA levels in the substantia nigra of healthy adult male rats, compared to controls (CON). Data are presented as mean ± SEM (n = 14/treatment group).

Gene expression levels of BDNF IV [*H*(_2_) = 0.881, *p* = 0.644] and its transcription factor NURR1 [*H*(_2_) = 1.663, *p* = 0.435] were unaltered in midbrain tissue homogenates obtained from normal adult rats chronically treated with typical or atypical antipsychotics for 7 months (Fig. 5a, b). However, the haloperidol treatment group had a non-significant mean increase in BDNF IV mRNA (41.19%) and NURR1 mRNA (46.46%) compared to controls, which is the opposite direction of change found in schizophrenia cases in the human post-mortem substantia nigra. Chronic antipsychotic treatment did not statistically change mRNA levels of BDNF receptors in the ventral midbrain, including TrkB^TK+^ [*H*(_2_) = 3.224, *p* = 0.199], TrkB^TK-^ [*H*(_2_) = 0.116, *p* = 0.944] and p75 [*H*(_2_) = 0.005, *p* = 0.998] (Fig. 5c–e).

### Gene expression correlations with demographic and clinical variables in the post-mortem human midbrain

BDNF IV, TrkB^TK+^ and NURR1 mRNAs positively correlated with RIN across the combined post-mortem cohorts used for qPCR (all *r* > 0.20; *p* < 0.05) (Table S8). TrkB^TK+^ (*r* = 0.209) positively and p75 (*r* = −0.235) mRNAs negatively correlated with PMI (*p* < 0.05) (Table S8) and most transcripts (4/5) correlated with pH, but in opposite directions with BDNF IV, TrkB^TK+^ positively correlated and TrkB^TK+^ and p75 negatively correlated with pH. As lowered pH is detected in the schizophrenia brain, pH is not considered an optimal covariate as it relates to our independent variable [59]. Further, pH and RIN are correlated, so the variance explained overlaps between these two variables. In our study, RIN was used as a covariate for BDNF IV, TrkB^TK+^ and NURR1 gene expression analysis between diagnostic and immune subgroups, and PMI was used as a covariate for TrkB^TK+^ and p75 mRNA analysis by diagnosis and immune subgroups. In the schizophrenia group, gene expression of BDNF IV (*r* = −0.347, *p* = 0.01) and NURR1 (*r* = −0.269, *p* = 0.036) negatively correlated with duration of illness and p75 mRNA positively correlated with daily CPZ-equivalent antipsychotic dose (*r* = 0.431, *p* = 0.045) (Table S9).

## Discussion

Reductions in BDNF in dopaminergic regions have previously been linked to dopamine dysfunction in vivo [62–64] and this is the first study to identify transcriptional changes reflective of reduced trophic support in the schizophrenia midbrain, with these alterations most profound in the presence of neuroinflammation. We find that reductions in trophic support proximal to midbrain dopamine neurons in schizophrenia include both the ligand and full-length receptor, with reduced BDNF and TrkB^TK+^ gene expression. We predict that these would also be present at the protein level, as is found in other brain regions in schizophrenia [27, 39]. Our finding of reduced NURR1 mRNA, especially in high inflammation schizophrenia cases, supports our hypothesis that these altered gene expression levels could impact the health and maintenance of dopamine neurons. In contrast, we find increases in putative “negative regulators” for BDNF both TrkB^TK-^ and p75 receptor mRNA. We also consider that these mRNAs are likely to be localised to astrocytes and oligodendrocytes, respectively, and therefore these increases may not directly impact BDNF signalling on midbrain dopamine neuronal cell membranes, but act indirectly. We posit that these putative changes in mRNA may impede neurotrophic signalling and could impact neurons, including dopamine neurons, of the substantia nigra.

Overall downregulation of BDNF mRNA in the schizophrenia group compared to controls in this study appears to be mostly found in cases in the high inflammatory subgroup, as corresponding reductions in BDNF mRNA are not present in schizophrenia cases with low inflammation. Lower levels of BDNF transcripts (I-IX and IV-IX) are also found in the DLPFC of schizophrenia cases with a high inflammatory biotype compared to those with a low inflammatory biotype [40], supporting that neurotrophin pathology may differ according to inflammatory status. Pro-inflammatory cytokines can compromise the capacity of BDNF to provide trophic support to neurons by interfering with signal transduction from the TrkB^TK+^ receptor to PI3K/Akt and Ras/ERK pathways, which are responsible for the neuroprotective effects of BDNF [65–67]. Unlike cytokines that are predominately expressed by glial cells, BDNF is mainly produced in neurons. In human midbrain, snRNAseq confirmed a primarily neuronal localization for BDNF expression in both low and high inflammation schizophrenia cases, as well as controls. However, further investigation into the specific subtypes of neurons where reductions in BDNF mRNA are localised in the schizophrenia midbrain is warranted.

Dysregulated transcription of BDNF in schizophrenia may be directly related to NURR1 as NURR1 is a transcription factor for the BDNF gene and NURR1 mRNA is also decreased in the midbrain in schizophrenia cases with high inflammation. NURR1 is critical for dopamine neuron survival as NURR1 deficiency and rare variants of the NURR1 gene contribute to neurodegeneration of dopamine neurons in Parkinson’s disease [68, 69]. Decreased NURR1 expression can precede downregulation of BDNF mRNA and is paralleled by reductions in TH in cultured mesencephalic neurons, which are less resilient to neurotoxicity with NURR1 deficiency [70], reflecting that there may be specific impediments to dopamine neuron survival in schizophrenia [71]. Hence, the observed reductions in NURR1 mRNA in the midbrain of high inflammation schizophrenia cases supports another feature of impaired trophic support for midbrain dopamine neurons and suggests compromised health of dopamine neurons in schizophrenia. However, the “health” status of dopamine neurons in the schizophrenia brain has not been well characterized to date.

Further evidence for disrupted BDNF-mediated trophic signalling in the midbrain are reductions in TrkB^TK+^ mRNA in both inflammatory schizophrenia subgroups compared to controls, demonstrating that reduced TrkB^TK+^ mRNA may be detected in the absence of overt tissue inflammation in schizophrenia. Using snRNAseq, we detected pan-TrkB (both TK+ and TK- splice variants) across multiple subtypes of neurons and astrocytes. This is consistent with the TrkB^TK-^ splice variant being principally localised to glial cells in the human DLPFC [16] and also supports that alterations in full-length TrkB mRNA can affect both dopaminergic and non-dopaminergic neurons in the midbrain. Indeed, BDNF and TrkB reductions are correlated with deficits in inhibitory neuron transcripts in the cortex of people with schizophrenia [25]. We have found evidence of GABA neuron dysfunction, marked by reductions in glutamate decarboxylase mRNA and protein, as well as reduced parvalbumin mRNA, in schizophrenia midbrain [72], suggesting these inhibitory neuron related deficits could also be correlated with neurotrophin reductions in subcortical regions as well as cortical regions. This reduction in TrkB^TK+^ mRNA could also impact dopamine neurons as TrkB hypomorphic mice with approximately 25–30% of normal levels of TrkB receptors display dopamine neuron loss in the nigrostriatal pathway [73], with accompanying gliosis [74]. We find levels of TrkB^TK+^ mRNA in schizophrenia are not as profoundly changed as in hypomorphic mice (~75% that of controls), yet this deficit could have a detrimental impact on midbrain dopamine neurons; however, a more extensive analysis of dopamine neurons in schizophrenia is needed.

In contrast to the full-length TrkB receptor, mRNA levels of the truncated receptor, TrkB^TK-^, were elevated in the midbrain of schizophrenia cases compared to controls, primarily in cases with high inflammation. BDNF binding to TrkB^TK-^ receptors on glia can promote the release of pro-inflammatory cytokines, including IL1β and TNFα, consistent with the increase of these cytokines at the mRNA and protein level we have found in the schizophrenia midbrain [66, 75–77]. We also find increased reactive astrocytes (GFAP) and microglia (IBA1) mRNAs in schizophrenia midbrain, and these cells are proposed mediators of the neuroinflammatory state in schizophrenia [9, 78]. Inflammatory activity of resident immune cells of the brain can also regulate neurotransmission as cytokines can evoke dopamine release and further perpetuate the hyperdopaminergic state of nigrostriatal dopamine neurons, and thereby may contribute to psychotic symptoms in schizophrenia [79, 80]. Altogether, the changes in BDNF, NURR1 and TrkB that are exacerbated in high inflammation schizophrenia cases may allude to greater disease severity in this subset of patients. Clinical studies report an interaction effect between blood levels of cytokines and BDNF in predicting cognitive performance [81, 82] and disease severity [83]. Here, we report that higher cytokines and lower BDNF co-occur in the midbrain of people with schizophrenia, supporting that this interaction is not just found in serum or plasma but extends to brain tissue.

Interestingly, increases in p75 receptor mRNA in schizophrenia midbrain appear to transcend inflammatory state and are found in both high and low inflammation subgroups of schizophrenia. This was an unexpected finding since p75 is upregulated in neurons and astrocytes in response to inflammation and oxidative stress in vitro [84, 85]. Also, in contrast to our expectations, snRNAseq of midbrain nuclei demonstrated that p75 receptor gene expression appears to be found mostly in oligodendrocytes in schizophrenia cases and controls. The p75 expression in oligodendrocytes was confirmed with immunohistochemistry; we found p75 receptor protein in oligodendrocyte-like cells and white matter tracts in many of the low and high inflammation schizophrenia cases, as well as in some controls. As p75 receptor expression in oligodendrocytes has a dual role, mediating both regenerative potential of these cells, but also promoting cell death [86, 87], these changes are difficult to interpret. While it is possible that increased p75 may be beneficial to oligodendrocytes in schizophrenia midbrain in the absence of neuroinflammation, we speculate that oligodendrocytes may be vulnerable to cell damage/death in high inflammation schizophrenia as mRNA for the cell-death ligand, TNFα, which is known to bind to p75, is also increased in schizophrenia midbrain. Indeed, myelin dysfunction, together with reductions in subcortical and cortical oligodendrocytes and associated transcripts, is found in schizophrenia in association with neuroinflammation [88–93]. Altogether, p75 receptor upregulation in the schizophrenia midbrain appears primarily localised in oligodendrocytes, indicating a role for the p75 receptor in contributing to white matter pathology in schizophrenia.

We have evidence that the transcriptional changes evident in midbrain BDNF, TrkB, p75 and NURR1 may not result from long-term antipsychotic exposure as we do not find any changes in these mRNAs in healthy adult rats chronically treated with either first- (haloperidol) or second-generation (risperidone) antipsychotic drugs. However, animal studies assessing the effects of chronic antipsychotic exposure with a shorter duration of treatment report reductions in BDNF in multiple brain regions [94–98]. Earlier studies implicated the mesolimbic pathway as the predominant site of therapeutic antipsychotic activity in reducing dopamine transmission [4, 99, 100], and we included cell bodies of both the nigrostriatal and ventral tegmental area in our studies, so any subregion specific effects of disease or antipsychotics on trophic support transcripts could be missed.

In humans, we find that midbrain p75 mRNA positively correlated with daily antipsychotic dose, supporting that p75 receptor upregulation may relate to greater disease severity and/or that increased p75 mRNA may be a consequence of antipsychotics. However, midbrain BDNF mRNA did not correlate with antipsychotic exposure, consistent with previous post-mortem studies which also report no significant correlations with lifetime antipsychotic doses [27, 28, 30]. Reductions in TrkB^TK+^ mRNA levels in the post-mortem human DLPFC in schizophrenia are found and correlate negatively with higher lifetime antipsychotic dose [15], so the potential impact of antipsychotics cannot be entirely ruled out. Gene expression of BDNF and NURR1 mRNA in the midbrain was found to negatively correlate with duration of illness in schizophrenia cases, and this may reflect that both mRNAs are known to decrease with age [101, 102], and the effect of increasing age on the brain may be more accelerated in schizophrenia [103, 104]. Furthermore, environmental factors such as childhood maltreatment may underlie BDNF deficits and may also contribute to immune priming [105]. Studies using a similar clustering approach as used here on pro-inflammatory mediators in blood show that high inflammation profiles are more common in schizophrenia patients with a history of childhood maltreatment [106, 107]. Additionally, early trauma in schizophrenia patients is linked to reductions in monocytes [108], increased pro-inflammatory markers [109, 110] and reduced BDNF levels [111, 112], reflecting a unique profile of changes associated with schizophrenia vulnerability that could stem from early developmental stages. Thus, stressful life events, increased inflammation and lower neurotrophins may all be linked in schizophrenia and may underpin a harsh microenvironment surrounding dopamine neurons. Future studies investigating the impact of early life stress on BDNF and neuroinflammation in schizophrenia with larger cohorts are warranted.

### Limitations

Post-mortem human schizophrenia brain tissue uniquely provides a snapshot of neuropathology in affected individuals; however, dynamic changes in inflammation cannot be ascertained in cross-sectional analysis. Additionally, metabolic and systemic alterations associated with cause of death and pre-existing comorbidities may also contribute to inflammatory profiles, many of which may not be captured in our study. The panel of inflammatory markers measured here was first identified from an unbiased RNAseq study of human cortical tissue, and after screening those cortically changed transcripts in midbrain tissue, a specific set of four markers was used to classify “high” and “low” subsets of individuals using midbrain measures. However, we recognize that within this group defined as “high inflammation”, the immune response in each individual is not likely the same. Indeed, inflammation may be triggered by multiple and distinct events and could involve activation of additional pathways or transcripts not assayed here. Thus, unique inflammatory subsets may exist within this “high inflammation” subgroup. Indeed, we recently analysed serum cytokines levels via machine learning and discovered that several inflammatory subgroups of people with schizophrenia could be identified [113, 114]. In our study, nuclei selected for snRNAseq analysis were mainly Olig2- suggesting that many cells that may express BDNF, TrkB and p75 were not selected for analysis. Additionally, subpopulations of dopaminergic neurons are mostly NeuN- in mammals [115] and would have not been selected for by our NeuN+ sorting step. Future snRNAseq analysis may instead select for cells that express a marker for dopamine neurons to specifically characterise the transcriptional landscape in midbrain dopamine neurons in schizophrenia. This study assessed the effects of chronic antipsychotic treatment on midbrain trophic transcripts in healthy adult rats; however, antipsychotics may differentially regulate trophic support molecules in the schizophrenia disease state, particularly when neuroinflammation is present. Additionally, the putative impact of PMI in human cohorts on transcripts assessed here were not modelled in our rodent study, where brains were collected rapidly in controlled conditions. Thus, it is possible that changes in transcript levels due to antipsychotics are only revealed with a delay in tissue harvesting and freezing. However, in our human studies only modest and inconsistent relationships between PMI and a minority of mRNAs were found.

## Conclusion

Here, we identified transcriptional reductions in beneficial neurotrophic factors in the schizophrenia midbrain, many of which are exacerbated by neuroinflammation and do not appear to be a direct consequence of chronic antipsychotic treatment. Increases in the truncated TrkB^TK-^ receptor may also compromise neurotrophic function in schizophrenia midbrain. Increased p75 receptor activity, likely in oligodendrocytes, in schizophrenia supports a role for the p75 receptor in these cells rather than altering trophic signalling within midbrain neurons. Further studies aiming to explore other hallmarks of dopamine neuron health, particularly in the presence of neuroinflammation, will inform therapeutic strategies to preserve cellular integrity and ameliorate potential functional deficits ensuing from the prospective loss of midbrain neurotrophic support in schizophrenia.

## Supplementary information

Supplemental Material

## Data Availability

All data generated or analysed during this study are included in this published article and its supplementary material. Data from snRNAseq analysis is available through the SMRI website www.stanleyresearch.org or directly at www.sncid.stanleyreserach.org.
