# Supplementary material for: Transcriptional evidence of reduced BDNF trophic capacity in the post-mortem human midbrain of schizophrenia cases with high inflammation
Source: Transl Psychiatry. 2025 May 7;15:162. doi: 10.1038/s41398-025-03359-7 (PMC12059047; doi:10.1038/s41398-025-03359-7)
Supplement: Supplementary file 1 — Supplemental Material [file 41398_2025_3359_MOESM1_ESM.docx]

**Supplementary Material**

**Table S1:** Detailed clinical demographics of cases from the Stanley Medical Research Institute and Tissue Resource Centre used for RT-qPCR analysis of post-mortem human midbrain tissue.

**Table S2:** Demographic and clinical variables for post-mortem human midbrain cases used for snRNAseq. *PMI = post-mortem interval*; *CPZ = Chlorpromazine equivalent dose (mg).*

| snRNAseq cohort | Control group (n=14) | Low inflammation schizophrenia group (n=10) | High inflammation schizophrenia group (n=10) | Statistics |
| --- | --- | --- | --- | --- |
| Age in years (range) | 44.7 ± 7.8 (34-60) | 40 ± 12.2 (19-53) | 42.7 ± 6.4 (33-54) | *F*_(2,32)_=0.804, *p*=0.46 |
| Sex (Male/Female) | 10/4 | 9/1 | 8/2 | — |
| pH | 6.6 ± 0.3 | 6.4 ± 0.3 | 6.5 ± 0.2 | *F*_(2,32)_=2.179, *p*=0.13 |
| PMI (hours) | 30.4 ± 12.2 | 23.3 ± 11.8 | 38.5 ± 18.1 | *F*_(2,32)_=2.909, *p*=0.07 |
| RIN | 6.0 ± 1.1 | 6.7 ± 1.5 | 6.3 ± 1.0 | *F*_(2,32)_=0.893, *p*=0.42 |
| Manner of death (Natural/Suicide) | 14/0 | 9/1 | 8/2 | — |
| Duration of illness (years) | — | 17.7 ± 9.6 | 21.9 ± 5.8 | — |
| Lifetime CPZ equivalent (mg) | — | 43986.1 ± 87972.3 | 126500 ± 94459 | — |
| Smoking around time of death | Yes=8, No=2, Unknown=4 | Yes=8, No=1, Unknown=1 | Yes=8, No=1, Unknown=1 | — |

**Table S3:** Demographic profiles of schizophrenia and control groups from the NSW Brain Tissue Resource Centre cohort used for immunohistochemistry.

| Immunohistochemistry cohort | Control group (n=27) | Schizophrenia group (n=27) | Statistics |
| --- | --- | --- | --- |
| Age in years (range) | 51.5 ± 11.9 (22-69) | 51.3 ± 11.7 (26-67) | *t*(54)= 0.081, *p*=0.94 |
| Sex (Male/Female) | 17/10 | 17/10 | — |
| pH | 6.6 ± 0.3 | 6.5 ± 0.2 | ***t*(54) = 2.015, *p=*0.049** |
| PMI (hours) | 33.0 ± 9.9 | 36.6 ± 18.5 | *t*(54)= -0.85, *p*=0.39 |
| Manner of death  (Natural/Suicide) | 27/0 | 22/5 | — |
| Duration of illness (years) | — | 29.3 ± 12.9 | — |
| Lifetime CPZ equivalent (g) | — | 8443.5 ± 8871.1 | — |
| Smoking around time of death | Yes=11, No=11, Unknown=5 | Yes=16, No=6, Unknown=5 | — |

### Group sizes vary as 6 cases (3 control, 3 schizophrenia) were excluded due to poor tissue quality. Data are presented as mean ± SEM. Bold test indicates statistically significant data.

**Table S4:** TaqMan gene expression assays used for RT-qPCR in the post-mortem human midbrain cohort.

| Gene | Transcript | TaqMan probe |
| --- | --- | --- |
| BDNF IV | Brain-Derived Neurotrophic Factor exon IV | Hs00380947_m1 |
| TrkB^TK+^ | Tropomyosin kinase B receptor with intracellular tyrosine kinase domain | Hs01093098_m1 |
| TrkB^TK-^ | Tropomyosin kinase B receptor (truncated) without intracellular tyrosine kinase domain | Hs01093110_m1 |
| p75 | Nerve growth factor receptor | [Hs00609976_m1](https://www.thermofisher.com/taqman-gene-expression/product/Hs00609976_m1?CID=&ICID=&subtype=) |
| NURR1 | Nuclear receptor-related factor 1 | Hs01118813_m1 |
| GUSB | β-glucuronidase | Hs00939627_m1 |
| GAPDH | Glyceraldehyde-3-phosphate dehydrogenase | Hs99999905_m1 |
| TBP | TATA-box binding protein | Hs00427620_m1 |
| UBC | Ubiquitin C | Hs99999903_m1 |

**Table S5:** TaqMan gene expression assays used for quantitative RT-qPCR in the rodent cohort.

| Gene | Transcript | TaqMan probe |
| --- | --- | --- |
| BDNF IV | Brain-Derived Neurotrophic Factor exon IV | Rn01484927_m1 |
| TrkB^TK+^ | Tropomyosin kinase B receptor with intracellular tyrosine kinase domain | Rn04338710_m1 |
| TrkB^TK-^ | Tropomyosin kinase B receptor (truncated) without intracellular tyrosine kinase domain | Rn01341751_m1 |
| p75 | Nerve growth factor receptor | [Rn00561634_m1](https://www.thermofisher.com/taqman-gene-expression/product/Hs00609976_m1?CID=&ICID=&subtype=) |
| NURR1 | Nuclear receptor-related factor 1 | Rn00570936_m1 |
| GUSB | β-glucuronidase | Rn00566655_m1 |
| GAPDH | Glyceraldehyde-3-phosphate dehydrogenase | Rn01775763_g1 |
| YWHAZ | Tyrosine-3-monooxygenase | Rn00755072_m1 |
| ACTB | β-actin | Rn00667869_m1 |

**Figure S1:** Gene expression of housekeeper genes **(a)** GUSB, **(b)** GAPDH, **(c)** TBP and **(d)** UBC and **(e)** the geomean of housekeepers is unchanged by diagnosis in the human post-mortem midbrain cohort (n=61 control, n=63 schizophrenia). All data are presented as mean ± SEM, with individual data points representing each case.

**Figure S2:** Gene expression of housekeeper genes **(a)** GUSB, **(b)** GAPDH, **(c)** YWHAZ and **(d)** ACTB and **(e)** the geomean of housekeepers is unchanged by treatment in the rodent cohort (n=14/group). All data are presented as mean ± SEM, with individual data points representing each sample.

**Table S6:** Top 10 transcripts used to define clusters of cells identified by snRNASeq of nuclei isolated from the midbrain of schizophrenia cases with low and high inflammation, as well as controls.

| Cluster | Cell type | Transcripts |
| --- | --- | --- |
| 0 | Microglia/  Macrophage 1 | APPB1IP, DOCK8, HS3ST4, LINC01374, KCNQ3, LNCAROD, P2RY12, SPP1, RUNX1 |
| 1 | Astrocyte 1 | GFAP, CD44, MIR43OOHG, HSPB8, RXF4, LINC01094, LINC01088, ADCY2, TTN, ADAMTSL3 |
| 2 | Astrocyte 2 | LINC00499, SLC1A2, HIPSE2, OI1-AS1, GPCS, SLC4A4, LINC00299, TNC, GLI3, NKAIN3 |
| 3 | Neuron 1 | GALNTL6, SPHKAP, NYAP2, SYNPR, OTX2-AS1, DLGAP1, NELL1, ZMAT4, GALNT14, LINC02055 |
| 4 | Microglia/  Macrophage 2 | P2RY12, DOCK8, APBB1IP, CX3CR1, LINC01374, LNCAROD, HS3ST4, KCNQ3, RUNX1, A2M |
| 5 | Neuron 2 | IQCJ-SCHIP1, RMST, ROBO1, CDH18, KCB2, HTR2C, KLHL1, FGF12, DAB1, PCDH15 |
| 6 | Astrocyte 3 | SLC1A2, LINC00499, HPSE2, DGKG, OBI1-AS1, SLC14A1, GPCS, LINC00299, GL13, TNC |
| 7 | Oligo 1 | ST18, MOBP, CTNNA3, MBP, RNF220, SLC5A11, PCSK6, MAN2A1, CDK18, SYNJ2 |
| 8 | Neuron 3 | NXPH1, CNTNAP2, ROBO1, DAB1, FGF12, GABRB3, CNTN5, GRIP1, DCC, ZNF804A |
| 9 | T-cell 1 | THEMIS, SKAP1, SLFN12L, BCL11B, PRKCH, ETS1, IL17R, CD247, PTPRC, ITGA4 |
| 10 | Oligo Neuron Mix | PLP1, TMEM144, SLC24A2, MBP, ST18, CTNNA3, LINC01608, PDE1C, RNF220, BCAS1 |
| 11 | Neuron 4 | TUBA1B, ATP1B1, VAMP2, SCG2, UCHL1, NEFL, NEFM, SV2A, NEFH, PEG10 |
| 12 | Fibroblast | CEMIP, LAMA2, ABCA9, BICC1, DCN, ARHGAP10, ARHGAP29, ABCA8, ATP1B3, ABCA10 |
| 13 | Microglia/  Macrophage 3 | F13A1, MRC1, CMAHP, MAN1A1, SELENOP, IQGAP2, CD163, SRGN, FMN1, RNF144B |
| 14 | Astrocyte 4 | HPSE2, LINC00499, SLC1A2, OBI1-AS1, SLC14A1, TNC, LINC02232, ITPRID1, ENTREP1, GPCS |
| 15 | Endothelial Cell | FLT1, ABCB1, ATP10A, MECOM, EPAS1, HERC2P3.1, ELOVL7, VWF, THSD4, PTPRB |
| 16 | Neuron 5 | RBFOX3, ZNF385D, CADPS2, CA10, CDH18, FSTL5, PDE10A, GRM4, CHN2, STXBP5L |
| 17 | Pericyte - smooth muscle cell | DLC1, RGS5.1, SLC38A11, GRM8, CARMIN, RM3, ATP1A2, MYO1B, EBF1, ITIH5 |
| 18 | Mix 1 | MEGF11, LHFPL3, TNR, VCAN, MMP16, SEMA5A, TMEM132C, SLC35F1, CA10, COL9A1 |
| 19 | Ependymal Cell | CFAP299, DNAH9, CFAP54, DCDC1, DNAH11, AGBL1, ZBBX, SPAG17, CFAP47, ARMC3 |
| 20 | Neuron 6 | CNTN5, FSTL4, LHFPL3, LRRC7, NTNG1, ADARB2, KIRREL3, IQCJ-SCHIP1, ATRNL1, FGF13 |
| 21 | Mesenchymal stem cell - lymphocyte | EYS, THSD4, GPC6, SLC4A4, SLIT2, CHSY3, C8orf34, PHLDB2, SLC38A4-AS1, GADL1 |
| 22 | Oligo2 | ST18, CTNNA3, PLP1, MBP, TMEM144, RNF220, MOBP, PDE1C, SLC24A2, EDIL3 |
| 23 | Neuron 7 | KCNQ5, NRG1, THSD7B, TAFA1, RBFOX1, RASGEF1B, CNTNAP2, FHOD3, ROBO2, DPP6 |
| 24 | Mesenchymal stem cell 1 | MTUS2, DCC, ZNF804A, SLIT2, EBF2, MCTP1, TENM1, CCSER1, GALNTL6, SORBS2 |
| 25 | Smooth muscle cell 1 | SLIT3, CRISPLD2, CARMN, RBPMS, ARHGAP10, RCAN2, COL4A1, DLC1, PTPRG, NR4A1 |
| 26 | Neuron 8 | KLHL11, SNTG1, KCNJ6, IQCJ-SCHIP1, GPC6, CADPS2, SGCD, PCDH15, CLSTN2, ROBO2 |
| 27 | Mix 2 (Neurons) | ADARB2, CNTN5, TMEM132D, GRIN2A, NXHP1, GRIP1, RELN, TMEM132B, PRELID2, NETO1 |
| 28 | T-cell 2 | THEMIS, SKAP1, TC2N, LINC00499, LINC01934, ETS1, BCL11B, CD247, ITGA4, STAT4 |
| 29 | Mesenchymal stem cell 2 | TAFA1, DGKB, GRM7, ARPP21, FGF12, ADARB2, MCTP1, ADGRL2, HS6ST3, GABRB2 |
| 30 | Neuron 8 | NRG1, ZNF385D, FGF12, ARPP21, HS6ST3, LINC01811, KCNH7, LHFPL3, DAB1, FSTL4 |
| 31 | Neuron 9 | CBLN2, DLGAP2, KCNQ5, ST6GALNAC5, GRIN2A, DAB1, RBFOX1, ADGRL2, MLIP, TMEM132D |
| 32 | Mix 3 (Neurons) | LINC01727, GRIA1, ADAMTSL1, SLC4A4, LINC02552, PLEKHH2, LINC01208, GRID2, ARHGEF28, LAMA2 |

**Table S7:** Pearson’s correlations for gene expression of BDNF correlates with receptor and transcription factor mRNA levels in the post-mortem human midbrain. Bold values indicate significant correlations between transcripts.

| Gene | Variable | TrkB^TK+^ | TrkB^TK-^ | p75 | NURR1 |
| --- | --- | --- | --- | --- | --- |
| BDNF IV | *n* | **109** | **109** | **105** | **108** |
|  | *r* | **0.563** | **-0.307** | **-0.362** | **0.419** |
|  | *p* | **<0.0001** | **0.001** | **<0.0001** | **<0.0001** |
| TrkB^TK-^ | *n* | 116 | - | 119 | 113 |
|  | *r* | -0.156 | - | -0.097 | -0.142 |
|  | *p* | 0.094 | - | 0.293 | 0.133 |
| p75 | *n* | **113** | 112 | - | **113** |
|  | *r* | **-0.362** | 0.055 | - | **-0.142** |
|  | *p* | **<0.0001** | 0.561 | - | **0.133** |
| NURR1 | *n* | **114** | 113 | **111** | - |
|  | *r* | **0.337** | -0.142 | **-0.232** | - |
|  | *p* | **<0.0001** | 0.133 | **0.014** | - |

**Table S8:** Pearson’s correlations for TrkB^TK+^, TrkB^TK-^, p75 and NURR1 mRNA and Spearman’s correlations for BDNF IV mRNA with demographic variables in the entire cohort, excluding 2SD outliers relative to diagnostic group. Bold values indicate significant correlations between genes and demographic variables.

| Gene | Variable | Age (years) | RIN | pH | PMI |
| --- | --- | --- | --- | --- | --- |
| BDNF IV | *n* | 112 | **112** | **112** | 112 |
|  | *r* | -0.15 | **0.426** | **0.413** | 0.17 |
|  | *p* | 0.113 | **<0.01** | **<0.01** | 0.131 |
| TrkB^TK+^ | *n* | 120 | **120** | **120** | **120** |
|  | *r* | 0.026 | **0.206** | **0.307** | **0.209** |
|  | *p* | 0.775 | **0.024** | **0.001** | **0.022** |
| TrkB^TK-^ | *n* | 120 | 120 | **120** | 120 |
|  | *r* | -0.057 | 0.006 | **-0.348** | -0.12 |
|  | *p* | 0.536 | 0.951 | **<0.01** | 0.191 |
| p75 | *n* | 117 | 117 | **117** | **117** |
|  | *r* | 0.141 | -0.112 | **-0.310** | **-0.235** |
|  | *p* | 0.130 | 0.189 | **0.001** | **0.011** |
| NURR1 | *n* | 118 | **118** | 118 | 118 |
|  | *r* | -0.17 | **0.260** | 0.139 | 0.012 |
|  | *p* | 0.066 | **0.004** | 0.134 | 0.899 |

**Table S9:** Spearman’s correlations between clinical variables and BDNF IV, TrkB^TK+^, TrkB^TK^, p75 and NURR1 mRNA levels in schizophrenia cases. Bold values indicate significant correlations between genes and clinical variables. *CPZ = Chlorpromazine equivalent dose (mg).*

| Gene | Variable | Illness Duration | Lifetime CPZ | Daily CPZ | Last Dose CPZ |
| --- | --- | --- | --- | --- | --- |
| BDNF IV | *n* | **54** | 48 | 16 | 22 |
|  | *r* | **-0.347** | -0.099 | 0.188 | 0.125 |
|  | *p* | **0.01** | 0.504 | 0.485 | 0.581 |
| TrkB^TK+^ | *n* | 61 | 55 | 20 | 26 |
|  | *r* | -0.133 | 0.149 | -0.182 | -0.171 |
|  | *p* | 0.305 | 0.279 | 0.442 | 0.403 |
| TrkB^TK-^ | *n* | 59 | 53 | 20 | 26 |
|  | *r* | 0.083 | 0.046 | -0.127 | -0.045 |
|  | *p* | 0.53 | 0.746 | 0.593 | 0.826 |
| p75 | *n* | 59 | 53 | **22** | 28 |
|  | *r* | 0.147 | 0.013 | **0.431** | -0.248 |
|  | *p* | 0.268 | 0.929 | **0.045** | 0.204 |
| NURR1 | *n* | **61** | 56 | 22 | 27 |
|  | *r* | **-0.269** | -0.242 | -0.304 | -0.16 |
|  | *p* | **0.036** | 0.072 | 0.17 | 0.426 |
